# Prediction Correction Topic Evolution Research for Metabolic Pathways of the Gut Microbiota

**DOI:** 10.3389/fmolb.2020.600720

**Published:** 2020-12-15

**Authors:** Li Ning, Peng Lifang, He Huixin

**Affiliations:** ^1^Management Science and Engineering Department, Management School, Xiamen University, Xiamen, China; ^2^Computer Science and Engineering Department, Computer Science and Engineering School, Huaqiao University, Quanzhou, China

**Keywords:** gut microbiota, metabolic pathway, LDA, time-series feature, topic prediction

## Abstract

The gut microbiota is composed of a large number of different bacteria, that play a key role in the construction of a metabolic signaling network. Deepening the link between metabolic pathways of the gut microbiota and human health, it seems increasingly essential to evolutionarily define the principal technologies applied in the field and their future trends. We use a topic analysis tool, Latent Dirichlet Allocation, to extract themes as a probabilistic distribution of latent topics from literature dataset. We also use the Prophet neural network prediction tool to predict future trend of this area of study. A total of 1,271 abstracts (from 2006 to 2020) were retrieved from MEDLINE with the query on “gut microbiota” and “metabolic pathway.” Our study found 10 topics covering current research types: dietary health, inflammation and liver cancer, fatty and diabetes, microbiota community, hepatic metabolism, metabolomics-based approach and SFCAs, allergic and immune disorders, gut dysbiosis, obesity, brain reaction, and cardiovascular disease. The analysis indicates that, with the rapid development of gut microbiota research, the metabolomics-based approach and SCFAs (topic 6) and dietary health (topic 1) have more studies being reported in the last 15 years. We also conclude from the data that, three other topics could be heavily focused in the future: metabolomics-based approach and SCFAs (topic 6), obesity (topic 8) and brain reaction and cardiovascular disease (topic 10), to unravel microbial affecting human health.

## Introduction

The microbiota—the collection of microorganisms that live within and on all mammals—provides crucial signals for the development and function of the immune system (Rooks and Garrett, 2016; Shin et al., 2017). The gut microbiota is composed of a variety of different bacteria which, produce plenty of compounds playing key roles in microbe selection and metabolic signaling network construction (Dodd et al., 2017). The gut microbiota is exclusively responsible to critical metabolic functions, including vitamin and short-chain fatty acid (SCFAs) production, amino acid synthesis, bile acid biotransformation, hydrolysis, and fermentation of non-digestible substrates (Putignani et al., 2016; Rowland et al., 2018; Strandwitz et al., 2019). The effects of gut microbiota include immune-cell homeostasis and development, epithelial homeostasis, enteric nerve regulation, support of angiogenesis, food digestion, and fat metabolism (Holmes et al., 2011; Zanni et al., 2017).

These effects are mediated by metabolites either produced by the microbes or derived from the transformation of environmental or host molecules. The gut microbiota is considered as a virtual endocrine organ that produces molecules which can interact with the host physiology and trigger responses at the local and distant levels (Zhang and Davies, 2016). Through the production/fermentation of metabolites, the gut microbiota regulates signaling pathways involved in intestinal mucosa homeostasis. When a balanced interaction between the gastrointestinal (GI) tract and resident microbiota is disrupted, intestinal, and extraintestinal diseases are prone to developing (Putignani et al., 2016), such as allergy, inflammatory bowel disease (IBD), obesity, cancer, and diabetes, metabolic disorders, cardiovascular dyslipidemia, and neuropathology (Holmes et al., 2011).

Despite the rapid development of Metabolic Pathways of the Gut Microbiota and its Metabolites in both the medical clinic and the academic world, few studies used natural language or bibliometric analyses to intuitively explore statistical relationships between titles, abstracts, and keywords in existing reports. Natural language processing can cut sentences into metadata, such as words, noun phrases, and so on. The bibliometric method can statistically analyze metadata according to the time and frequency of occurrence. The resulted outcome can then be visualized using image processing, so that the evolution of research topics in Gut Microbiota are intuitively displayed along with time. In brief, combining the analysis results with the time series calculation method is likely to provide enlightening views for the development and prediction of research hotspots at the technical perspective.

In recent years, scientific production on the metabolic pathways of gut microbiota and its metabolites have produced datasets of extreme great interests and has expanded the physiology field of microbiome concepts. It is increasingly necessary to recognize emerging links, both theoretically and empirically (Poeker et al., 2019). This is momentous for the comprehensive assessment of gut Microbiota metabolism pathways. A serviceable way to accomplish that goal is to evaluate the frequency of emerged scientific terms and how the same terms are aggregated in research on personality and their disorders (Deng et al., 2014). Latent Dirichlet Allocation, a statistical technique, used in the present study, allows us to grasp and explain potential conceptual connections between terms that appear at a higher frequency in recent scientific products through a layered aggregation system of words (Blei et al., 2003). Hence, compared with traditional bibliometric research paper, this article is characterized by natural language processing of a large number of title texts and predictive analysis based on time series, as well as a visual analysis of subject results based on the predictive modification.

In the perspective, the scientific terms presented in the published abstracts on MEDLINE over the last 15 years were summarized. We extract the research topics and calculated the theme intensity from these titles and abstracts. The theme intensity was predicted based on the time segment of the month. From the perspective of natural language processing (NLP) and bibliometric analysis, our study provides specific methods for scientific researchers in this field, and furnishes more followers with intuitive understanding and disciplinary analysis in the study of intestinal diseases and metabolic pathways of intestinal microbiology. The method adopted in this paper has several advantages. The thematic model used in this paper describes and predicts the evolution of time segments, which is beneficial for scholars to track the development of research and to look for opportunities for expansion. The results beyond theoretical and framework recognition can facilitate scholars and readers to understand the evolutionary trends of research topics in the field of gut microbiota.

## Methods

This study evaluates the “Gut Microbiota” and “Metabolic Pathways” to explore the evolution of the research topic. The research framework is mainly embodied in two stages: the first phase is data preprocessing and topic extraction. The purpose of this stage is to represent. The intensity of each topic has time-series features. Thus, the second phase concentrates on the prediction analysis and display of the topic trend. The aim of this stage is to predict topic development and discover more possibilities for hot research content. The first stage serves as the foundation and premise of the research. The second one plays a part in the purpose and result of the study.

### Dataset

This article explored and recovered titles, abstracts, and keywords from publications regarding “Gut Microbiota” and “Metabolic Pathways” using Web of Knowledge-MEDLINE database. The MEDLINE database is produced by the Us National Library of Medicine and covers a wide range of biomedical disciplines.

We used the following keywords to extract literatures from the MEDLINE database: gut microbiota [TS] AND metabolic pathway [TS] AND “2006/01/01 [DATE]”: “2020/08/01 [DATE].” Information retrieved includes title, author, abstract, keywords, references, and journal sources. In total, 3,588 literature were obtained. We then filtered the literatures to only keep journal articles for downstream analysis, which leaves 1,651 non-duplicated literatures. Topic distributions of studies between 2006 and 2020 (Appendix 1), suggest that studies on gut microbiota metabolic pathways have continued growing over the past 5 years. To further analyze the development trend of the extracted research topic, 1,271 observed literature titles, abstracts, and keywords were further processed by natural language routine.

### Topic Modeling

Latent Dirichlet allocation (LDA) was proposed by Blei in 2003, on which a layer of Dirichlet conjugating prior distribution was added based on the PLSI model (Hofmann, 1999; Blei et al., 2003). In recent years, scholars in the field of microbiology and molecular biosciences have used the LDA model and its improved algorithm to identify scientific research topics and applied it to intelligence analysis based on text data (Qiu and Yu, 2018; Mehari et al., 2019). In addition to using a corpus (Li et al., 2019), topic modeling can also use additional information, such as time stamp and author network (Rosen-Zvi et al., 2010; Xu et al., 2020), as prior variables or observation variables. Other variants are by embedding new potential variables such as emotions, or by adding constraints applicable to a corpus from a specific scenario (Wang et al., 2018).

Existing topic discovery and evolution techniques are mainly developed based on LDA probabilistic topic model. LDA describes is a three-tier Bayesian network (Blei et al., 2003). For the evaluation of the effect of topic models, perplexity is a standard tool to measure the effect of language models in natural language processing. We use the perplexity and coherence index to be the guideline for the selection of the number of topics. *log_perplexity* and *model.coherence* are used to calculate the results. The lower the perplexity, the better the effect of the topic number, and the better the training subject distribution model can fit the training set data. When the number of topics increases, the perplexity of titles, and abstracts decreases gradually. The coherence of the topic tends to be stable gradually after reaching the optimal level. When *K* is 10, the title and abstract topic reaches it's optimum and tends to be stable (Appendix 2). Therefore, we set the number of topics *K* to 10.

### Topic Intensity

After topic extraction, we calculate the topic intensity. Topic intensity is a statistical attribute of the topic itself, which is used to represent the degree of concern of the topic. In our research, the number of documents distributed to each topic is used as the calculation of intensity. Define in *c* time slice, in the document set, the number of documents is *Dc*, and the intensity of topic *Z* can be defined as the number of articles attributed to topic *Z*:

$$\begin{array}{l} ST_{z}=\underset{d\in D_{c}}{\sum }\theta_{dz} \end{array}$$

The intensity of each topic forms a time series. If a month is taken as a time slice, the incomplete data time slice is first removed and the previous data is used for modeling. However, at the same time, the data in the latest time slice can reflect the topic trend in the latest time, so the data in this time slice is used for prediction and correction, and the future trend prediction is made based on the model and predicted data.

### Prediction Correction Model

At present, the commonly used time series model includes ARIMA, LSTM neural network, and so on. The former is more effective in a short time series, while the latter is more effective in long time series. In our study, the Prophet neural network prediction tool will also be used. Compared with the classical ARIMA model, the Prophet model can better predict the growth trend. It does not require large sample data for text training, so it is easier to achieve convergence than the LSTM method. Since the minimum unit for the journal publication cycle in the data sample is monthly. When predicting the size trend of each topic, we take a month as the unit and temporarily ignore the influence of cycle factors and holiday factors.

We use RMSE and R2_score to measure the deviation between the observed value and the real value and evaluate the quality of the trend prediction results. RMSE represents the root mean square error; R2_score calculates the goodness of fitting, R2 value ranges from 0 to 1, the closer R2 is to 1, the better the fitting effect is. To investigate whether the development trend of the research topic is linear or not, we compare between Prophet-Liner and Prophet-Logistic results. Prophet-Liner Trend represents the direct prediction result of linear Trend on the original data, and Prophet-Logistic Trend indicates the direct prediction result of Logistic Trend on the original data.

### Topic Trend

Since the collection time of articles corresponding to all research topics is the same, in the final time slice, it can be approximately assumed that the missing proportion of each research topic is the same. According to this missing proportion, the value predicted by using the Prophet model is further been modified. Finally, based on the forecast-modified topic size, the topic intensity evolution trend can be calculated for the future period.

**Figure 1:**
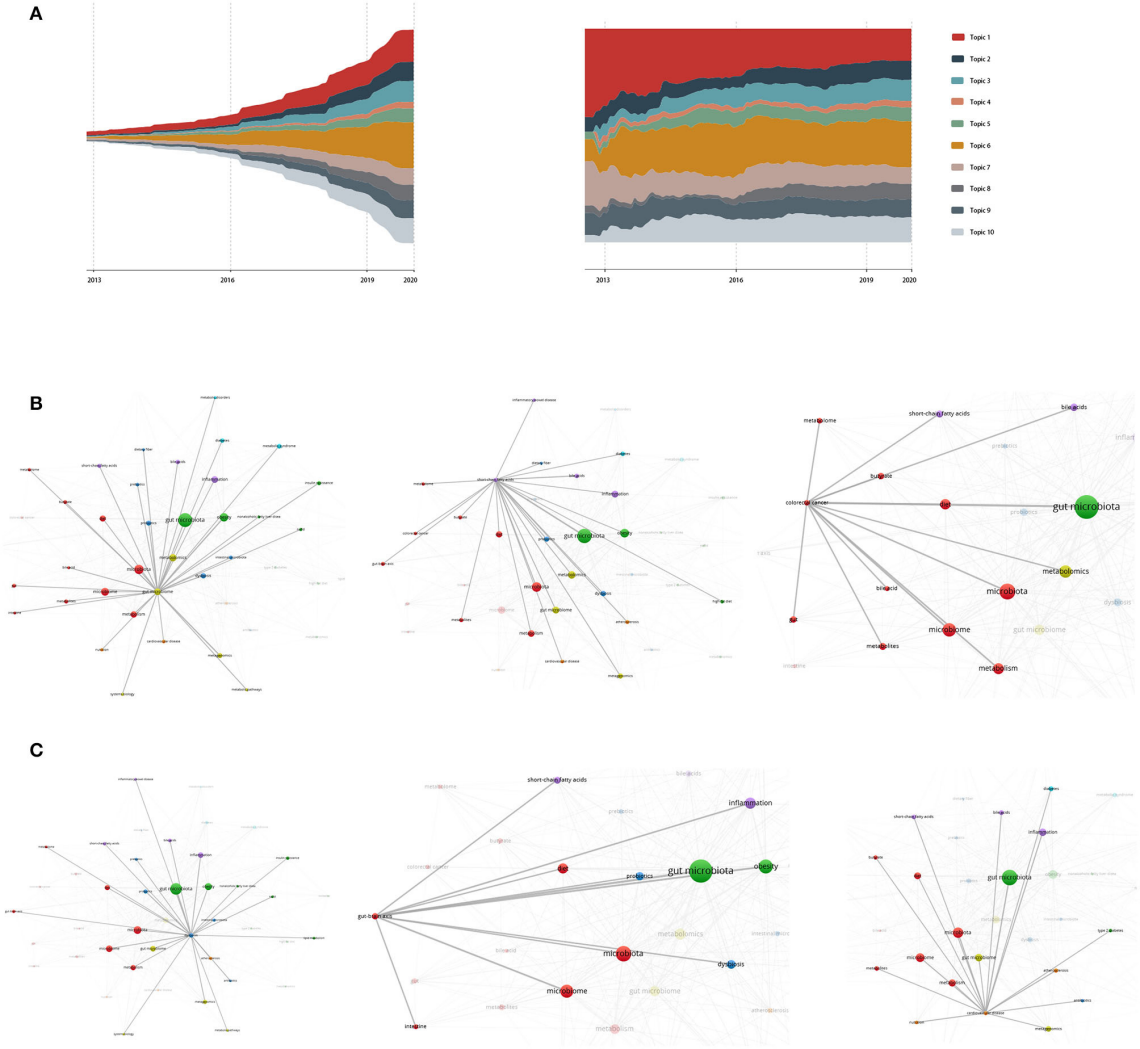
**(A)** is the river map and river scale map of the predicted-modified topic intensity evolution trend. Topic 1 dietary health, 2 inflammation and liver cancer, 3 fatty and diabetes, 4 gut microbiota community, 5 hepatic metabolism, 6 metabolomics-based approach and SFCAs, 7 allergic and immune disorders, 8 gut dysbiosis, 9 obesity, 10 brain reaction and cardiovascular disease. **(B–D)** show keyword co-occurrences of high frequency words in Topic 6, 8, and 10.

We use the ThemeRriver diagrams in ECharts series to represent changes in events or topics over time. The different colored strip river branches in the theme river encode different events or topics, and the width of the river branch encodes the value in the original dataset. In addition, the time attributes in the original dataset are mapped to a single timeline. Figure 1 shows the strength of the evolution trend of research topics for metabolic pathways of the gut microbiota and its metabolites. Each color represents 1 of the 10 topics extracted; the width of the chromaticity represents the different intensity of the topic. Based on the predicted-modified results, we predict the cumulative number of documents assigned to the end of 2020.

To discover the relationship for subjects in each topic, we draw a co-occurrence map of keywords using VOSviewer. Nodes are clustered by topics. The size of nodes in the figure reflects the frequency of occurrence of this keyword, and the color of nodes represents clustering. The correlation threshold was set as 7 (Jeyaraj and Zadeh, 2020), that is, if two keywords appeared together and more than six literature produced correlation. Of 2,557 keywords, 76 meet the threshold. The darker the color of the line between nodes, the higher the degree of correlation between nodes.

## Results

### Topic Extractions

Among the keywords shown in most topics, disease, intestinal, microbiome, metabolite, and microbiota have the highest frequency. However, the recognition degree for these words is relatively low, making it difficult to explain the detailed research questions in the topic. Therefore, in the following results for topic extraction, we only list the top 10 words with the highest frequency in each topic group with terms “microbiota” and “metabolism” etc. excluded due to they are less informative in our analysis. Topic names are ranked by their frequency as shown in Table 1. In summary, these topics include: dietary health (174 studies); inflammation and liver cancer (122 studies); fatty and diabetes (140 studies); gut microbiota community (46 studies); hepatic metabolism (97 studies); metabolomics based approach and SCFAs (266 studies); allergic and immune disorders (104 studies); gut dysbiosis (88 studies); obesity (108 studies); and brain reaction and cardiovascular disease (127 studies). Column 5 in Table 1 summarizes the weights of each topic, indicating the numbers of documents that are assigned to the corresponding topics. Among the topics, Topic 6 holds the most assigned documents, indicating the increased research trends in SCFAs, and CC. To explore whether each topic meets their development stages, a forecast to the number of documents in the topic is also performed to infer possible evolution trends of topics till December 31, 2020.

**Table 1:** Results for topic extraction and topic prediction.

	**Most probable word**	**RMSE**	**R2_score**	**Topic %**	**Predicted %**
		**(ARIMA, Prophet-liner; Prophet-logistic)**			
Topic 1 Dietary health	Health, dietary, interaction, pathway, response, induce, disease, regulation, receptor, and gastrointestinal	(367.42, 27.38, 28.06)	(0.8665, 0.9652, 0.9979)	13.7	14.97
Topic 2 Inflammation and liver cancer	Intestinal, inflammation, liver, cancer, associate, alter, liquid, murine, infection, and potential	(259.36, 36.88, 15.93)	(0.4752, 0.9394, 0.9961)	9.6	8.97
Topic 3 Fatty and diabetes	Fatty, expression, diabetes, liver, composition, serum, diversity, alcoholic, signaling, and epithelial	(192.51, 53.27, 17.56)	(0.3723, 0.9448, 0.994)	11.0	9.89
Topic 4 Gut microbiota community	Community, control, perturb, response, glucose, metabolomics, clinical, feeding, environment, and homeostasis	(101.39, 12.72, 4.86)	(0.4168, 0.9131, 0.9693)	3.6	3.04
Topic 5 Hepatic metabolism	Pathway, induce, bacterial, composition, hepatic, function, antibiotic, link, amino, and probiotic	(159.25, 42.62, 10.70)	(0.3982, 0.9072, 0.9853)	7.6	6.50
**Topic 6 Metabolomics based approach and SFCAs**	**Metabolomics, cancer, reveal, fatty, acid, chain, short, colorectal, dysbiosis, and disorder**	(334.91, 31.46, 38.83)	(0.9496, 0.9747, 0.9967)	20.9	21.61
Topic 7 Allergic and immune disorders	Lactobacillus, immune, modulate, bifidobacterium, innate, cell, butyrate, commensal, inhibit, and autism	(152.70, 32.52, 15.42)	(0.6409, 0.9376, 0.9958)	8.2	7.70
**Topic 8 Gut dysbiosis**	**Dysbiosis, mechanism, therapeutic, target, immune, probiotic, crosstalk, allergy, treatment, and kynurenine**	(244.05, 18.94, 12.81)	(0.8647, 0.9248, 0.9911)	6.9	7.27
Topic 9 Obesity	Dietary, obesity, modulate, potential, trimethylamine, oxide, compound, lipid, ameliorate, and resistance	(298.00, 25.74, 14.36)	(0.6151, 0.9330, 0.9911)	8.5	8.40
**Topic 10 Brain reaction and cardiovascular disease**	**Brain, cardiovascular, bowel, inflammatory, interaction, tryptophan, system, protective, functional, and heart**	(155.00, 31.58, 21.70)	(0.9035, 0.9551, 0.9971)	10.0	11.65

### Trend Intensity Prediction

Column 3 and 4 in Table 1 demonstrates the contradistinctive results of three Trend prediction methods: ARIMA time series analysis model, Prophet model (liner trend) and Prophet model (logistic trend). Two conclusions can be observed. First, for each research topic, the *R*^2^ value of the Prophet model exceeds 0.90, indicating that the Prophet model can well-fit the evolution trend of the research topic. The reason is that the distribution of each topic has an obvious growth trend, the sequence is non-stationary, so the effect of the ARIMA model is poor. The Liner growth model in the Prophet model is by the growth model in the field of gut microbiota metabolic pathways. Prophet model has fewer parameters, so it is easy to achieve a better prediction effect. Second, the time evolution rules of various research topics are inconsistent, and most of the research topics are more in line with the Liner trend. The reason is that most of the topics in this field are in the period of rapid growth and have not yet reached saturation growth. However, the Logistic trend fitting effect of Topic 6 is better, which is slightly better than the Liner model, showing that this Topic has experienced rapid growth in the past few years. Currently, topic 6 receives higher attention and higher recognition.

Column 6 in Table 1 shows the predicted results on December 31, 2020, based on the Prophet predicted-modified results. It can be found that the research achievements for various topics explored exhibited an overall growth trend. But the growth trends in each field are slightly different. Compared between Column 5 and 6 in Table 1, the weights of Topic 1, 6, 8, and 10 are higher than their original ratio. However, the remaining topic trends show decreases. It means that by the date of December 31, 2020, there will be more journal documents related to Topic 1, 6, 8, and 10. To further explain the prediction results, the correlation between keywords with the highest word frequency in each subject will be discussed.

### Trend Analysis

We examined the topic words of the other six topics and found that the co-occurrence of most of the subjects did not reach the threshold of 5, which explains that the contribution of most of the subjects was relatively low. Therefore, the link strength of topic words in Topic 2, 3, 4, 5, 7, and 9 was relatively low. Besides, most words in Topic 1 are more macroscopic and less recognizable, so we will discuss Topic 6, 8, and 10 in their keyword network. In detail:

Topic 6 refers to the importance of Metabolomics as a key meta-omics approach and SCFAs as a key element to study the modulation of the gut microbiota. The co-occurrence of SCFAs and Metabolomics increased by over 70 and 150 times during the last 15 years. SCFAs are the microbial metabolites implicated in colorectal cancer, and other metabolic and neurological disorders, such as Kidney disease or Central nervous system dysfunction (Kim et al., 2016; Barton et al., 2018). Although, there is a strong association between SCFAs, colorectal cancer, disorders, and dysbiosis, the links between metabolomics and SCFAs are to be uncovered. Clinical studies demonstrated that the administration of SCFAs has a positive effect on the treatment of ulcerative colitis and obesity, bowel disorders, and cancer (Cong et al., 2018). Thus, more research using a metabolomics-based approach to unravel bacterial metabolism should be dugout.

Topic 8 focuses on the importance of research on dysbiosis as a shift to healthy gut microbiota. The co-occurrence of dysbiosis has increased by 36 times. Low bacterial gene counts have been associated with altered gut microbial dysbiosis and have been linked to increased insulin resistance, obesity, and the metabolic syndrome (Boursier et al., 2016; Putignani et al., 2016). Individuals with these characteristics are more likely to develop metabolic diseases. That is to say, there is a strong association between dysbiosis, probiotics, inflammation, and cardiovascular disease that makes topic 8 grow rapidly.

Topic 10 describes brain reaction and human diseases due to allergic and immune disorders or metabolic disorders. The word brain rarely appears as a keyword on its own. The keyword related to the “brain” is the gut-brain axis. In addition to the co-occurrence of the gut-brain axis and cardiovascular disease shown in Figure 1, the co-occurrence of inflammatory bowel diseases has increased by more than 20 times. Inflammatory bowel disease (IBD) is associated with changes in the gut microbiota, characterized by a decreased abundance of Clostridia and an overall reduction in bacterial diversity (Bonfili et al., 2017; Santoru et al., 2017). Therefore, as the keyword dysbiosis mentioned in Topic 8, increasing clinical research will be discovered to link dysbiosis with various immune and metabolic disorders in intestinal sites.

## Discussion and Conclusion

### Conclusions

The scientific panorama involved in the study of the metabolic pathways of gut microbiota and its metabolites on human health and human disease is described in the 10 latent topics. Among the 1,271 sample studies, keyword metabolomics shows 155 co-occurrences and 80 links. In addition to the word “microbio” or “metaboli” stems, the core element from the current scientific discussion is the metabolomics-based approach. For each research topic, we forecast the evolution trend of topics based on time series features. Prophet model can better adapt to the evolution trend. The Liner growth model in the Prophet model is by the growth model in the field of gut microbiota metabolic pathways. The time evolution rules of various research topics are inconsistent, and most of the research topics are more in line with the Liner trend. It is hoped that the research methodology proposed in this study will be reflected in academic and clinical practice to promote a different approach to conceptualizing and treating gut microbiota metabolic pathways based on existing methodologies.

### Discussions

From the analysis of the data, it is demonstrated that the metabolomics-based approach and SCFAs (topic 6) and dietary health (topic 1) have been more focused during the last 15 years. Based on our analysis, it is clear that metabolomics-based approach and SCFAs (topic 6), obesity (topic 8), brain reaction and cardiovascular disease (topic 10) will be paid more attention in the near future, in adherence with the quantitative analysis of all small molecular metabolites in cells of a biological system at a given time and under certain conditions. In the study of health and disease in gut microbes and the metabolic pathways of their metabolites, the topic of microbial communities remains rare evolved in the near future. In our opinion, this area of investigation requires a much greater commitment than that described by our study, which identified 46 studies in this area in topic 4.

Our analysis in this perspective summarizes the existing evolution and predicts the future trends of researches in Metabolic Pathways of the Gut Microbiota. In the first place, the topic model used in this paper describes and predicts the evolution of time segments, which does good for scholars to track the research development and find opportunities to expand it. Second, the results go beyond the recognition of theory and framework and help scholars and readers to understand the hot spots and future topic combination in the study of gut microbiota metabolic pathways.

It is noted that we also limited our analysis in the following aspects. Firstly, although we described the hot research issues and predicted the frontier research issues through natural language processing technology and bibliometric methods, there is still a dearth of standard in the natural language. Besides, the rules of language vary in different areas of medical research. So far, some basic rules can be found, but one root can express many levels of meaning, or the same meaning can be expounded in many ways. This paper tries harder to comprehend natural language based on rules, but still has a few deviations. Secondly, the conclusion of this paper does not involve to find out the research deficiencies, technical challenges, and other problems in the field of metabolic pathways of gut microbiota and its metabolites on human health and human disease. As a result, these deficiencies need to be further solved optimizing algorithms and developing professional language dictionaries or expert systems in the future for the sake of providing a more comprehensive analysis for the research problems.

## Data Availability Statement

The original contributions presented in the study are included in the article/Supplementary Materials, further inquiries can be directed to the corresponding author/s.

## Author Contributions

LN wrote the manuscript. HH contributed to the modification of the manuscript. PL also contributed to the modification of the manuscript. All authors contributed to the article and approved the submitted version.

## Conflict of Interest

The authors declare that the research was conducted in the absence of any commercial or financial relationships that could be construed as a potential conflict of interest.
