# Supplementary material for: Prediction Correction Topic Evolution Research for Metabolic Pathways of the Gut Microbiota
Source: Front Mol Biosci. 2020 Dec 15;7:600720. doi: 10.3389/fmolb.2020.600720 (PMC7793741; doi:10.3389/fmolb.2020.600720)
Supplement: Supplementary file 1 [file Data_Sheet_1.docx]

**Appendix**


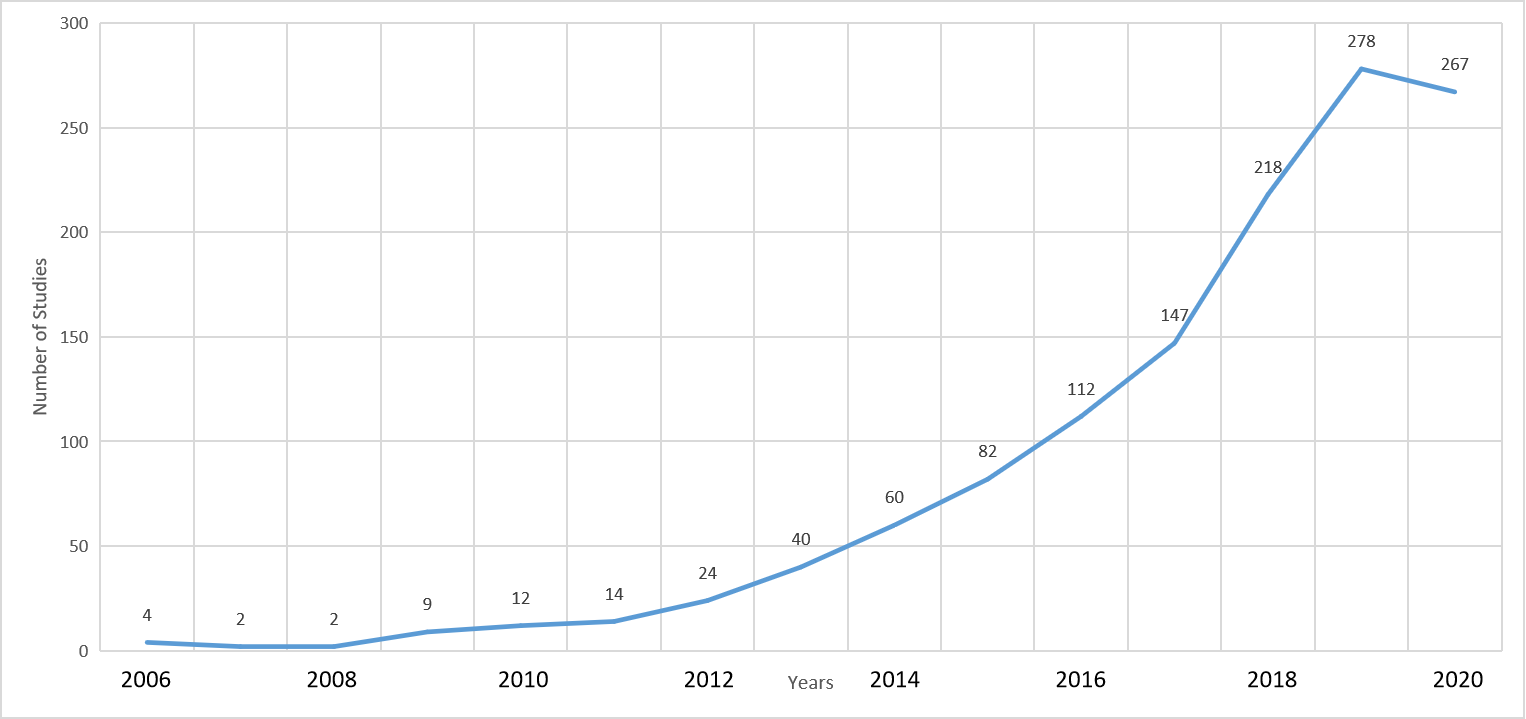


**Appendix 1 Distribution of relevant studies over time (according to MEDLINE)**

**Appendix 2 Perplexity and Coherence for the number of topics**$\boldsymbol{K}$
